# Behenic acid alleviates inflammation and insulin resistance in gestational diabetes mellitus by regulating TLR4/NF-κB signaling pathway

**DOI:** 10.1016/j.isci.2024.111019

**Published:** 2024-09-24

**Authors:** Kerong Liu, Ying Gu, Xingnan Pan, Sha Chen, Jie Cheng, Le Zhang, Minkai Cao

**Affiliations:** 1 Department of Endocrinology, Affiliated Children’s Hospital of Jiangnan University (Wuxi Children’s Hospital), Wuxi 214023, Jiangsu, China; 2 Department of Obstetrics and Gynecology, Affiliated Women’s Hospital of Jiangnan University (Wuxi Maternity and Child Health Care Hospital), Wuxi 214002, Jiangsu, China; 3 Department of Pediatric, Affiliated Children’s Hospital of Jiangnan University (Wuxi Children’s Hospital), Wuxi 214023, Jiangsu, China; 4 Center for Reproductive Medicine, Renji Hospital, School of Medicine, Shanghai Jiao Tong University, Shanghai 200135, China; 5 Shanghai Key Laboratory for Assisted Reproduction and Reproductive Genetics, Shanghai 200135, China; 6 Department of Neonatology, Affiliated Children’s Hospital of Jiangnan University (Wuxi Children’s Hospital), Wuxi 214023, Jiangsu, China

**Keywords:** Molecular biology, Endocrinology of pregnancy, Laboratory endocrinology

## Abstract

Gestational diabetes mellitus (GDM) is a distinct form of diabetes that poses a significant threat to the health of both pregnant women and fetuses. The objective of this study was to investigate the impact of behenic acid (BA) on glucose metabolism, inflammation, and insulin resistance in GDM mice, and to elucidate the underlying molecular mechanism. Here, we demonstrated that daily administration of 10 mg/mL BA during pregnancy effectively ameliorated abnormal glucose metabolism in GDM mice and their offspring and improved birth outcomes in the offspring. Moreover, BA promoted the proliferation of islet β cells, restored their normal function, and augmented glucose uptake by skeletal muscle cells. Mechanistically, BA mitigated inflammation and insulin resistance in GDM mice by inhibiting activation of the TLR4/NF-κB signaling pathway. Our study provides compelling evidence supporting the efficacy of BA in improving GDM, suggesting its potential use as a dietary supplement for preventing and treating GDM.

## Highlights

• BA improves glucose metabolism in GDM mice and enhances offspring birth outcomes
• BA promotes proliferation of islet β cells and restores their normal function
• BA enhances glucose uptake in skeletal muscle cells to maintain insulin sensitivity
• BA mitigates inflammation and insulin resistance by inhibiting TLR4/NF-κB pathway

## Graphical abstract

**Figure:**
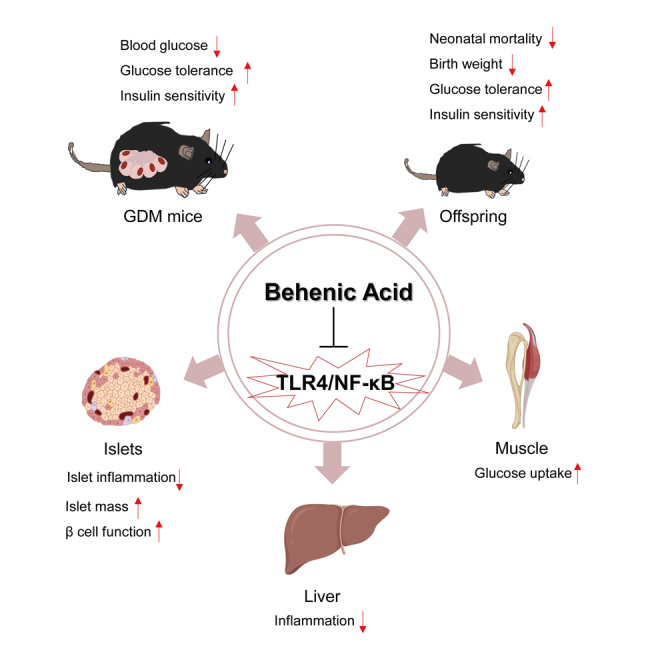

## Introduction

Gestational diabetes mellitus (GDM) is a distinct form of diabetes mellitus characterized by abnormal glucose tolerance that is initially diagnosed during pregnancy, encompassing both pre-existing abnormal glucose metabolism that was first detected during pregnancy and newly developed abnormal glucose tolerance during pregnancy.^1^ GDM can lead to various adverse outcomes in pregnant women and fetuses, including dystocia, infection, habitual abortion, neonatal congenital malformations, and perinatal mortality.^2^ The symptoms of GDM are typically subtle and self-awareness is rare, posing challenges for independent detection. Most individuals exhibit abnormal glucose tolerance between 24 and 28 weeks of pregnancy.^3^^,^^4^ Currently, it is widely accepted that the placenta secretes various hormones during pregnancy, leading to diminished insulin sensitivity and inadequate insulin secretion, ultimately contributing to the development of GDM.^5^^,^^6^ Most pregnant women with GDM can gradually achieve normoglycemia postpartum; however, research indicates that more than half of the patients will develop type 2 diabetes mellitus (T2DM) within 5–10 years after delivery.^6^ Furthermore, the offspring of GDM patients are also affected in terms of heightened susceptibility to obesity and T2DM, as well as an increased risk of chronic conditions such as cardiovascular disease.^7^ Recent studies have revealed a correlation between GDM and cognitive impairment, attention deficit, and other nervous system impairments in the offspring.^8^ Consequently, the prevention and management of GDM have emerged as a significant global public health concern.

It is widely recognized that diet plays a pivotal role in the development of diabetes.^9^ Alterations in lipid intake can result in changes in fatty acid levels within the body, thereby affecting human health.^10^ Consequently, some scholars have proposed that maternal nutrition management represents a potential and safe approach to prevent GDM, with fatty acids identified as one of the key nutrients.^11^^,^^12^ Despite numerous studies investigating the relationship between fatty acids and diabetes, no consistent conclusions have been reached. The diverse structures and functions of fatty acids may play distinct roles in the development of GDM.^13^ In the past, it was generally believed that saturated fatty acids (SFA) were detrimental to human health. However, an increasing body of research has demonstrated that different subtypes of SFA have varying impacts on human health.^14^ Studies have demonstrated a positive correlation between the levels of long-chain even SFA (including C14:0, C16:0, and C18:0) and the development of diabetes, hypertension, and atherosclerosis. Conversely, even SFA (including C20:0, C22:0, and C24:0) exhibit the potential to mitigate the occurrence of metabolic disorders, such as diabetes, cardiovascular disease, and cancer.^15^^,^^16^ Behenic acid (BA) (C22:0) is a natural SFA with an ultra-long chain and an even number of carbon atoms that can be found in various plants. It possesses antioxidant, anti-inflammatory, antibacterial, and antitumor properties and has been shown to be a natural inhibitor of pancreatic lipase, which is effective in suppressing the development of obesity.^17^^,^^18^ Several epidemiological studies have demonstrated that circulating BA concentrations are negatively correlated with the incidence of T2DM and may act as a free fatty acid receptor 1 (FFAR1) agonist to stimulate insulin secretion.^19^^,^^20^ However, whether BA can be used to improve GDM remains unclear.

The pathogenesis of GDM is highly intricate. Current research predominantly posits that GDM development is related to insulin resistance (IR) and chronic inflammation.^21^ Toll-like receptor (TLRs) family members play a pivotal role in the formation of chronic inflammation and IR.^22^ Recent studies have shown that TLR4-mediated chronic inflammation induced by the maternal innate immune system during pregnancy can aggravate IR in pregnant women and eventually lead to GDM.^23^^,^^24^^,^^25^ Lipopolysaccharide (LPS) is a typical exogenous ligand of TLR4.^26^ Endogenous ligands for TLR4 primarily include free fatty acids, elevated glucose levels, and heat shock proteins. It is widely acknowledged that these endogenous ligands can activate TLR4-mediated inflammatory pathways independent of exogenous ligands, such as LPS.^27^^,^^28^ Nuclear factor kappa B (NF-κB), a downstream signaling molecule of TLRs, is essential for the immune response.^29^ There is compelling evidence that activation of the TLR4/NF-κB signaling pathway is related to the pathogenesis of IR and diabetes.

This study aimed to explore the effects of BA on GDM in mice, focusing on its impact on inflammation, islet β-cell function, and skeletal muscle glucose uptake, as well as its potential to alleviate IR and inflammation through the TLR4/NF-κB signaling pathway. This study presents a safe and effective strategy for GDM prevention and treatment.

## Results

### BA alleviates insulin resistance and glucose tolerance in GDM mice

**Figure 1:**
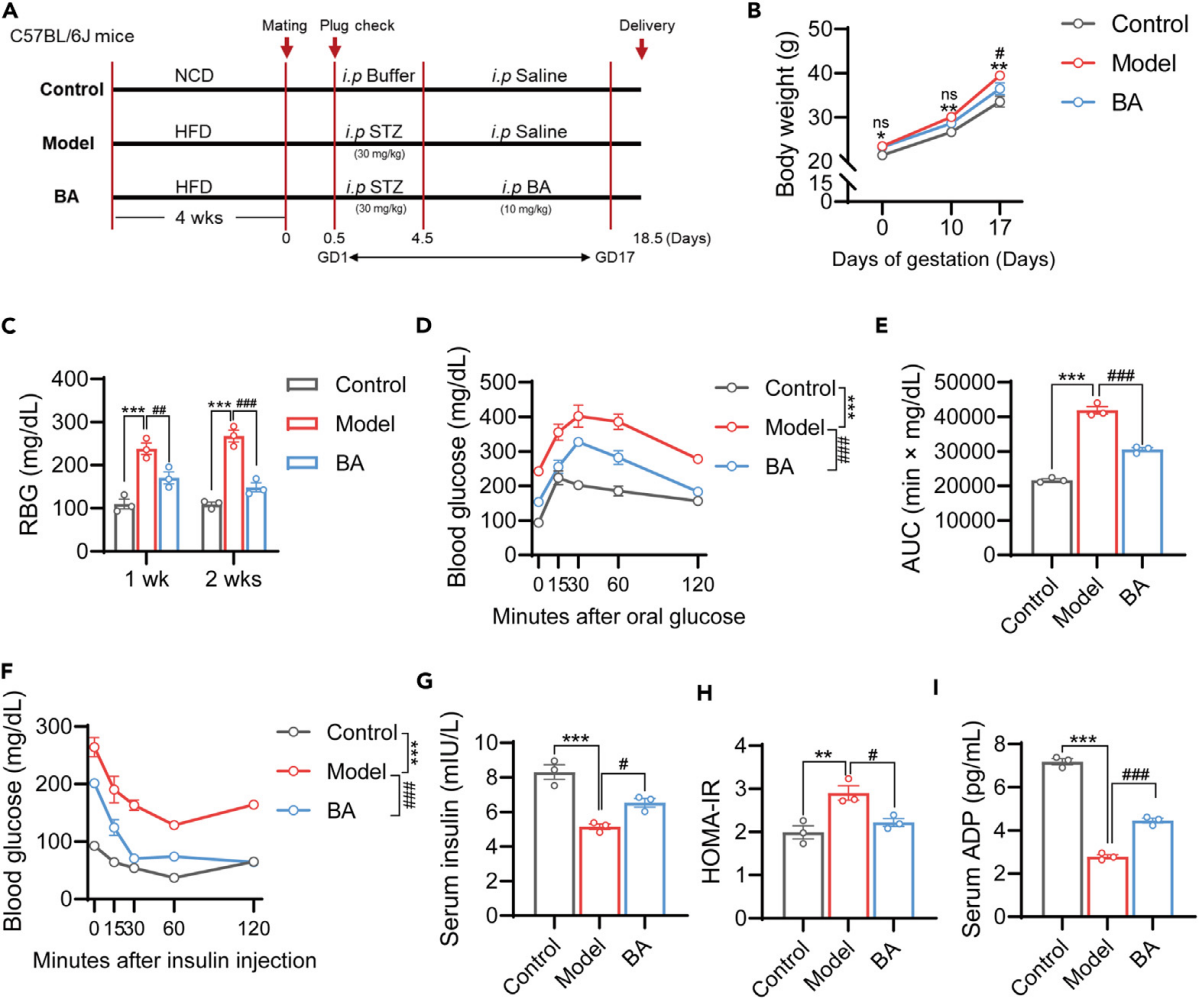
BA alleviates insulin resistance and glucose tolerance in GDM mice (A) Schematic representation of the experimental animal program. (B) Body weights of mice examined before and 10 and 17 days after gestation. (C) Random blood glucose (RBG) was measured in the Control, Model and BA group mice after 1 and 2 weeks of treatment. (D and E) Oral glucose tolerance test (OGTT) curve (D) and area under the curve (AUC) (E) on GD14. (F) Insulin tolerance test (ITT) curve at GD16. (G) Serum insulin concentrations of mice in the three groups were measured on GD17. (H) HOMA-IR was calculated based on measured fasting blood glucose and insulin concentrations. (I) The concentration of serum adiponectin (ADP) was measured in the three groups of mice. Data represented as means ± SEM. *n* = 3 mice per group. Unpaired two tailed t test for (B), One-way ANOVA for (C, E and G–I), two-way ANOVA for (D and F). ∗*p* < 0.05, ∗∗*p* < 0.01, ∗∗∗*p* < 0.001 vs. Control/Model group. ^#^*p* < 0.05, ^##^*p* < 0.01, ^###^*p* < 0.001, ns, not significant. vs. Model/BA group. See also Figure S1.

To elucidate the role of the ultra-long-chain fatty acid BA in the pathogenesis and progression of GDM, we established a mouse model of GDM using a short-term high-fat diet (HFD) combined with low-dose STZ injection. In the absence of established dosage guidelines from prior research, we administered BA intraperitoneally to adult female mice at doses of 0, 5, 10, 15, and 20 mg/mL for 15 consecutive days. The results showed that higher concentrations (15 and 20 mg/mL) exerted a notable impact on hepatic and renal functions in mice (Figures S1A–S1D). Consequently, a dose of 10 mg/mL was selected for the treatment of GDM mice, which demonstrated efficacy without adversely affecting liver and kidney function (Figure 1A). Compared to the model group, BA intervention reduced the body weight of the mice by approximately 2 g prenatally (Figure 1B). After 1 and 2 weeks of treatment, the random blood glucose levels in the model group were significantly elevated; however, BA treatment effectively reduced the random blood glucose levels in GDM mice (Figure 1C). OGTT was conducted to analyze the impact of BA therapy on glucose homeostasis, and the results revealed that BA administration enhanced the clearance of glucose in GDM mice (Figures 1D and 1E). Additionally, an ITT assay was conducted to assess alterations in the insulin response of the GDM mice (Figure 1F). Following BA administration, a significant enhancement was observed in the insulin response of the GDM mice. Additionally, a low insulin level was observed in the model group, which was restored by the administration of BA (Figure 1G). Furthermore, we evaluated the IR among the groups and found that HOMA-IR was elevated in the model group, whereas BA treatment ameliorated IR (Figure 1H). Moreover, BA treatment increased ADP levels in GDM mice (Figure 1I). These findings suggest that BA can improve glycolipid metabolism in GDM mice.

### The administration of BA improves the birth outcome of the offspring of GDM mice

**Figure 2:**
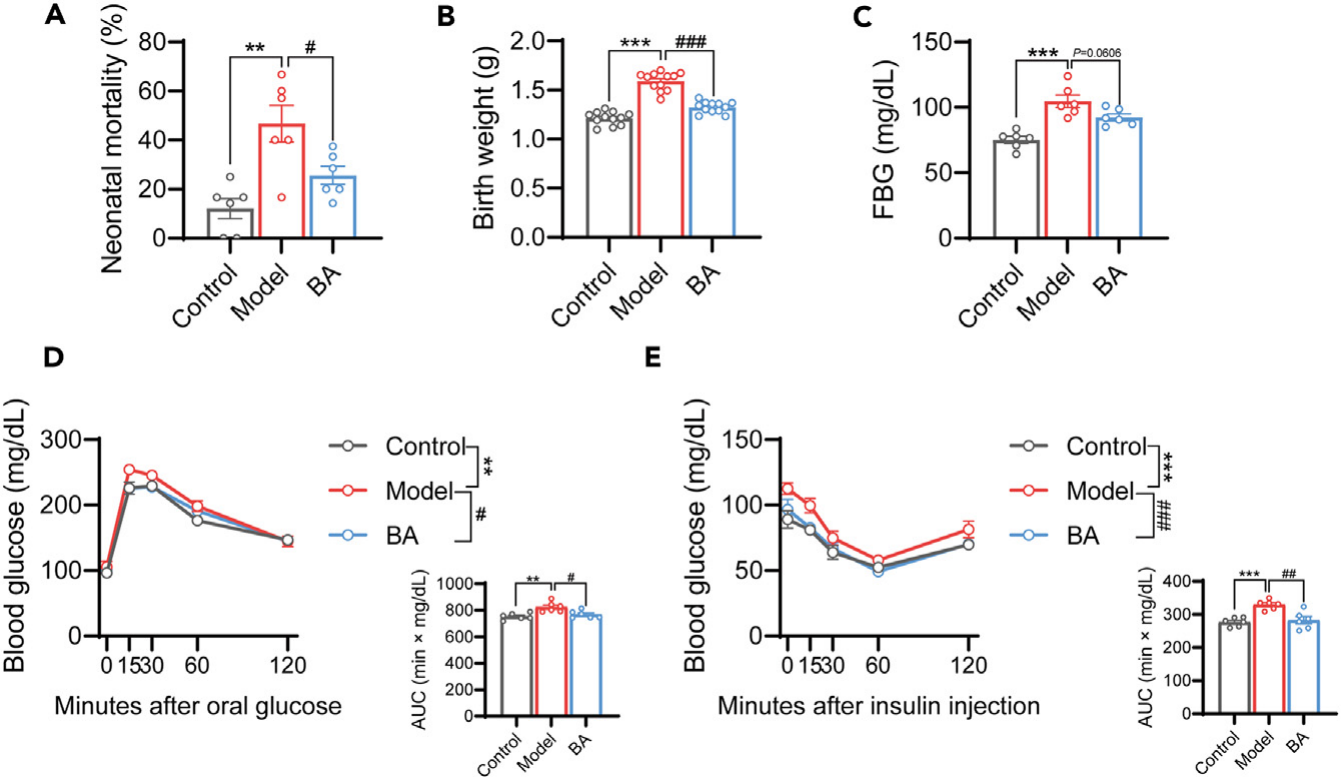
The administration of BA improves the birth outcome of the offspring of GDM mice (A) The mortality of the offspring of mice in the control, model, and BA groups was recorded and compared. *n* = 6. (B) Birth body weights of offspring mice were measured. *n* = 12. (C) Fasting blood glucose (FBG) concentrations in offspring were measured at three weeks. *n* = 6. (D) Oral glucose tolerance test (OGTT) curve and area under the curve (AUC) of mice at three weeks. *n* = 6. (E) Insulin tolerance test (ITT) curve and AUC under the curve of the mice at 3 weeks *n* = 6. Data represented as means ± SEM. One-way ANOVA for (A–C) and two-way ANOVA for (D and E). ∗∗*p* < 0.01, ∗∗∗*p* < 0.001 vs. the Control/Model group. ^#^*p* < 0.05, ^##^*p* < 0.01, ^###^*p* < 0.001 vs. Model/BA group. See also Figure S2.

GDM can also lead to various adverse consequences in offspring. Therefore, we investigated the effects of BA on the offspring of GDM mice. Compared with the model group, the BA-treated group exhibited significantly reduced mortality (Figure 2A), and decreased birth weight of the pups (Figure 2B). Furthermore, BA treatment contributed to a decrease in fasting blood glucose levels in the offspring of GDM mice (Figure 2C). Additionally, our study revealed that offspring of BA-treated mice displayed improved glucose clearance (Figure 2D) and enhanced insulin responsiveness (Figure 2E). We analyzed females and males separately and found no sex differences in the effects of BA on birth outcomes in offspring (Figure S2). These results suggest that BA has potential benefits in improving birth outcomes in GDM mouse progeny.

### BA preserves islet mass in GDM mice

**Figure 3:**
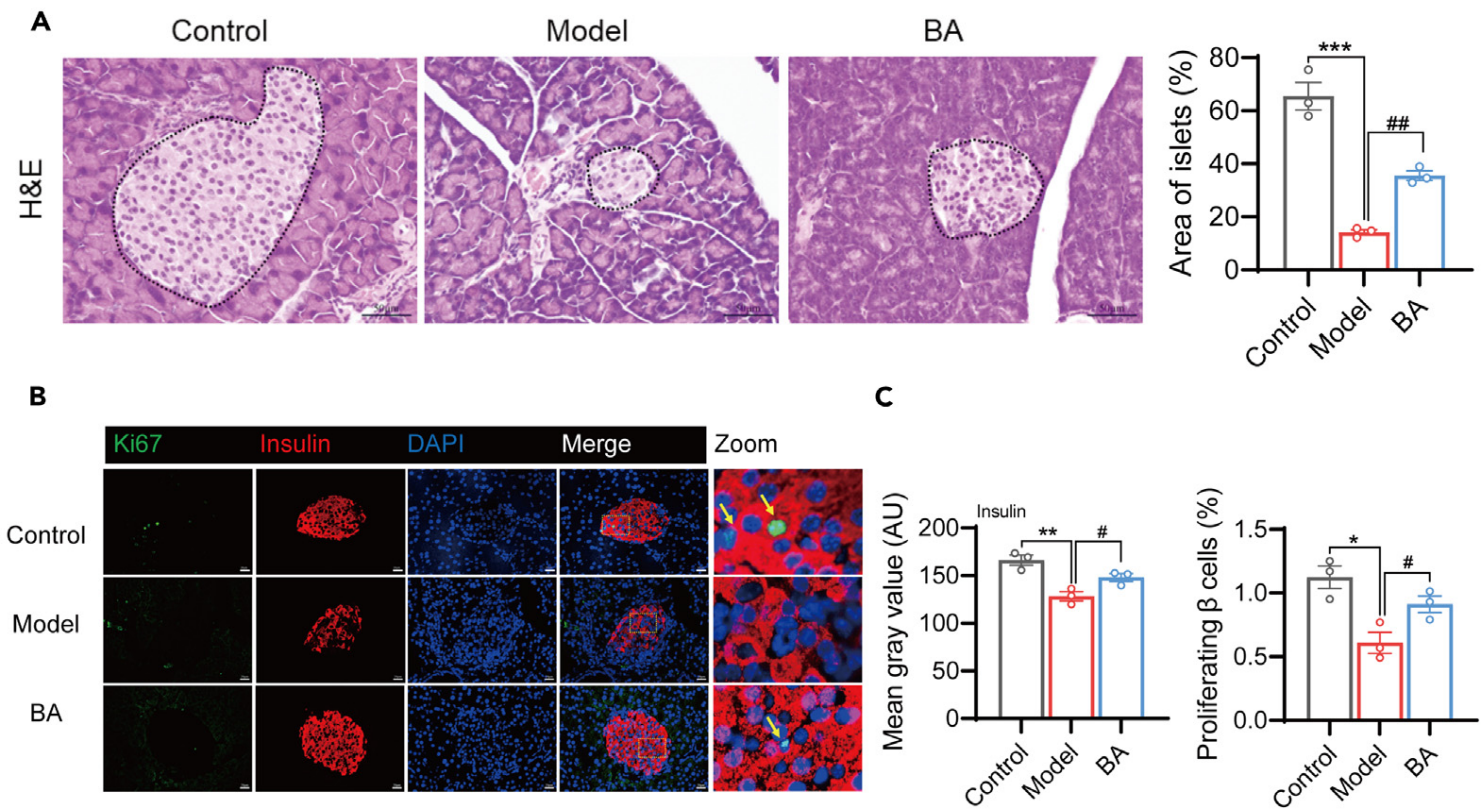
BA preserves islet mass in GDM mice (A) H&E staining of pancreatic tissues of mice in the control, model, and BA groups was performed to evaluate the number and volume of the islets. Scale bar, 50 μm. (B and C) Immunofluorescence staining was conducted to observe the insulin-positive and Ki-67 positive islet β cells in pancreatic tissues (B), quantification of insulin fluorescence intensity and ki67-positive pancreatic β cells as a percentage of β cells (C). Arrows indicate proliferating β-cells. Scale bar, 20 μm; zoom is a 5× enlargement of the original image. Data represented as means ± SEM. *n* = 3 mice per group. One-way ANOVA. ∗*p* < 0.05, ∗∗*p* < 0.01, ∗∗∗*p* < 0.001 vs. Control/Model group. ^#^*p* < 0.05, ^##^*p* < 0.01 vs. Model/BA group.

Next, we investigated the protective effects of BA on islet β cells. Histological examination of pancreatic tissue using H&E staining revealed a significant reduction in both the number and volume of islets in GDM mice, which improved after BA treatment (Figure 3A). Immunofluorescence staining demonstrated a substantial increase in the population of insulin-positive β cells within the pancreas after BA treatment compared with that in the model group. To further validate that BA can promote the proliferation of islet β cells, immunofluorescence staining of Ki67 in mouse islet β cells was performed. The results demonstrated a significant increase in the proportion of Ki67-positive β cells following BA treatment in GDM mice (Figures 3B and 3C). These histological analyses provide evidence that BA exerts protective effects on the islets of GDM mice and promotes islet β cell proliferation.

### BA attenuates systemic inflammation by inhibiting pro-inflammatory cytokines and chemokines secretion

**Figure 4:**
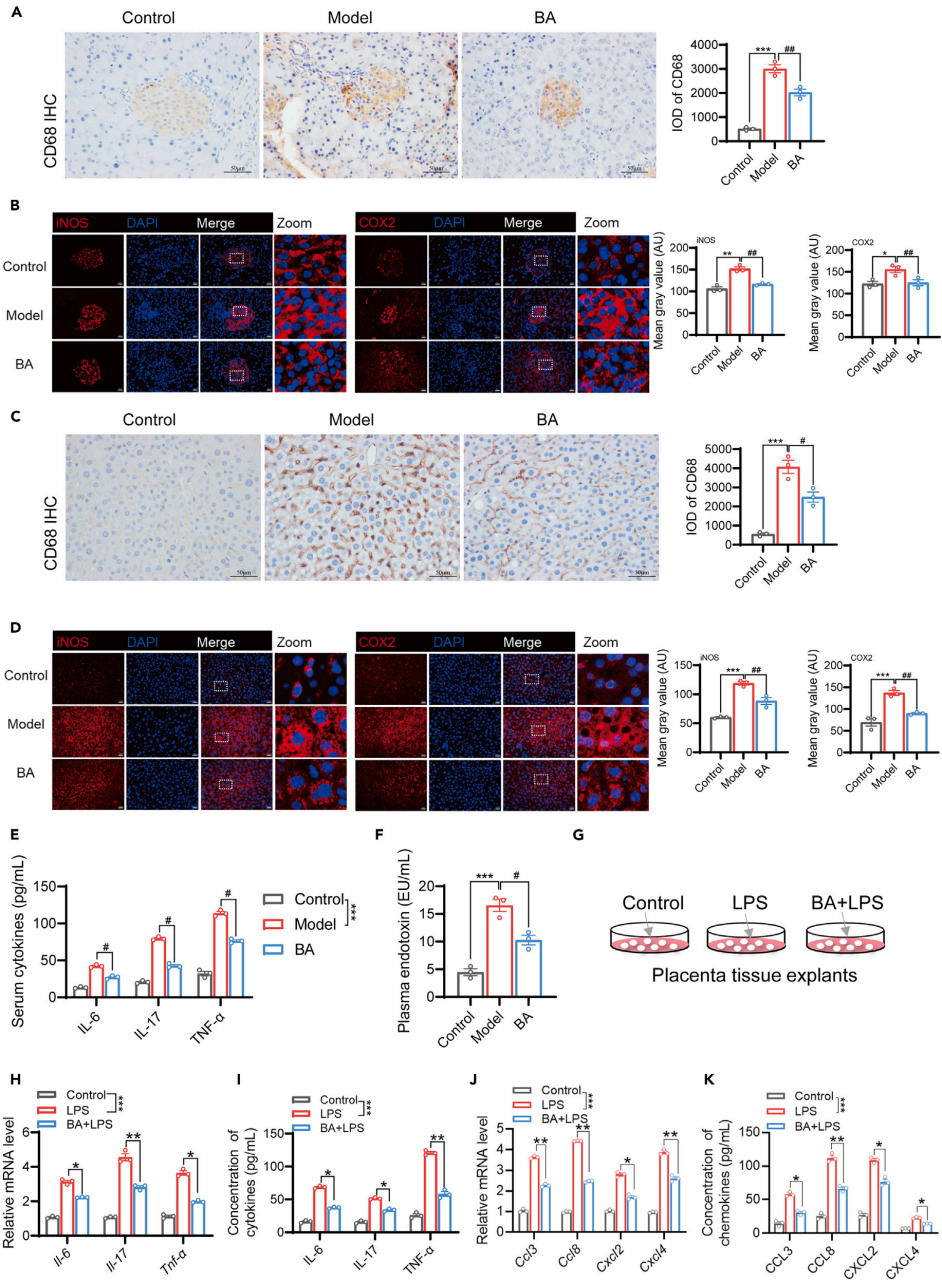
BA attenuates systemic inflammation by inhibiting pro-inflammatory cytokines and chemokines secretion (A) The expression of CD68 in the pancreas of mice in the control, model, and BA groups was assessed by IHC on GD17. Scale bar, 50 μm. The integrated optical density (IOD) level of CD68 was shown on the right. (B) Expression of pancreatic iNOS and COX2 was examined by immunofluorescence staining in GD17 mice. Scale bar, 20 μm and zoom is a 5x enlargement of the original image. Quantification of mean fluorescence intensity was shown on the right. (C) The expression of CD68 in the liver tissues of mice in the control, model, and BA groups was assessed by IHC on GD17. Scale bar, 50 μm. The integrated optical density (IOD) level of CD68 was shown on the right. (D) Expression of hepatic iNOS and COX2 was examined by immunofluorescence staining in GD17 mice. Scale bar, 20 μm and zoom is a 5x enlargement of the original image. Quantification of mean fluorescence intensity was shown on the right. (E and F) Serum concentrations of pro-inflammatory cytokines IL-6, IL-17, and TNF-α were measured by ELISA (E) and plasma endotoxin was evaluated using the chromogenic kinetic method (F) in GD17 mice. (G) Experimental scheme: Pregnant mice placental tissue explants were constructed and stimulated with LPS (10 μg/mL) alone for 24 h or pre-treatment with BA for 1 h. Detection of cellular and supernatant expression of inflammatory factors and chemokines. The tissues and supernatants were assayed for inflammatory and chemokine expression. (H) Relative mRNA expression of pro-inflammatory cytokines Il-6, Il-17, and Tnf-α was detected by RT-qPCR. (I) Secretion of IL-6, IL-17, and TNF-α in the medium was assessed by ELISA. (J) Relative mRNA expression of chemokines Ccl3, Ccl8, Cxcl2, and Cxcl4 was detected by RT-qPCR. (K) Concentrations of CCL3, CCL8, CXCL2, and CXCL4 secreted in the medium were determined by ELISA. Data represented as means ± SEM. *n* = 3 per group. One-way ANOVA. ∗*p* < 0.05, ∗∗*p* < 0.01, ∗∗∗*p* < 0.001. ^#^*p* < 0.05, ^##^*p* < 0.01 vs. Model/BA group.

We also investigated the potential anti-inflammatory effects of BA. IHC staining revealed a significant reduction in CD68-labeled macrophage infiltration in the pancreas of GDM mice following BA treatment (Figure 4A). Using immunofluorescence staining, we observed that the two inflammation-related proteins iNOS and COX2 were highly expressed in the pancreatic tissue of GDM model group mice. Their expression levels were reduced in the BA-treated groups (Figure 4B). Liver macrophage infiltration and inflammatory factor expression were also upregulated in GDM mice compared to those in control mice, which was attenuated by BA treatment (Figures 4C and 4D). In addition, after BA treatment, the serum concentration levels of the pro-inflammatory cytokines IL-6, IL-17, and TNF-α were significantly reduced compared to those observed in the model group of GDM mice (Figure 4E). Furthermore, our findings demonstrated a significant elevation in plasma endotoxin levels in GDM model mice, whereas treatment with BA effectively reduced endotoxin content (Figure 4F). To further explore the inhibitory effect of BA on inflammation, we established an *in vitro* cell model by stimulating the placental tissue with LPS (Figure 4G). It was evident that the mRNA expression and secretion of the proinflammatory cytokines IL-6, IL-17, and TNF-α were significantly upregulated following LPS treatment. However, BA treatment effectively suppressed mRNA expression and secretion (Figures 4H and 4I). Additionally, BA suppressed the mRNA expression and secretion of chemokines CCL3, CCL8, CXCL2, and CXCL4, which are closely associated with the inflammatory response (Figures 4J and 4K). These findings suggest that BA can attenuate the inflammatory response in GDM by inhibiting the secretion and expression of pro-inflammatory cytokines and chemokines.

### BA restores skeletal muscle glucose uptake to maintain insulin sensitivity

**Figure 5:**
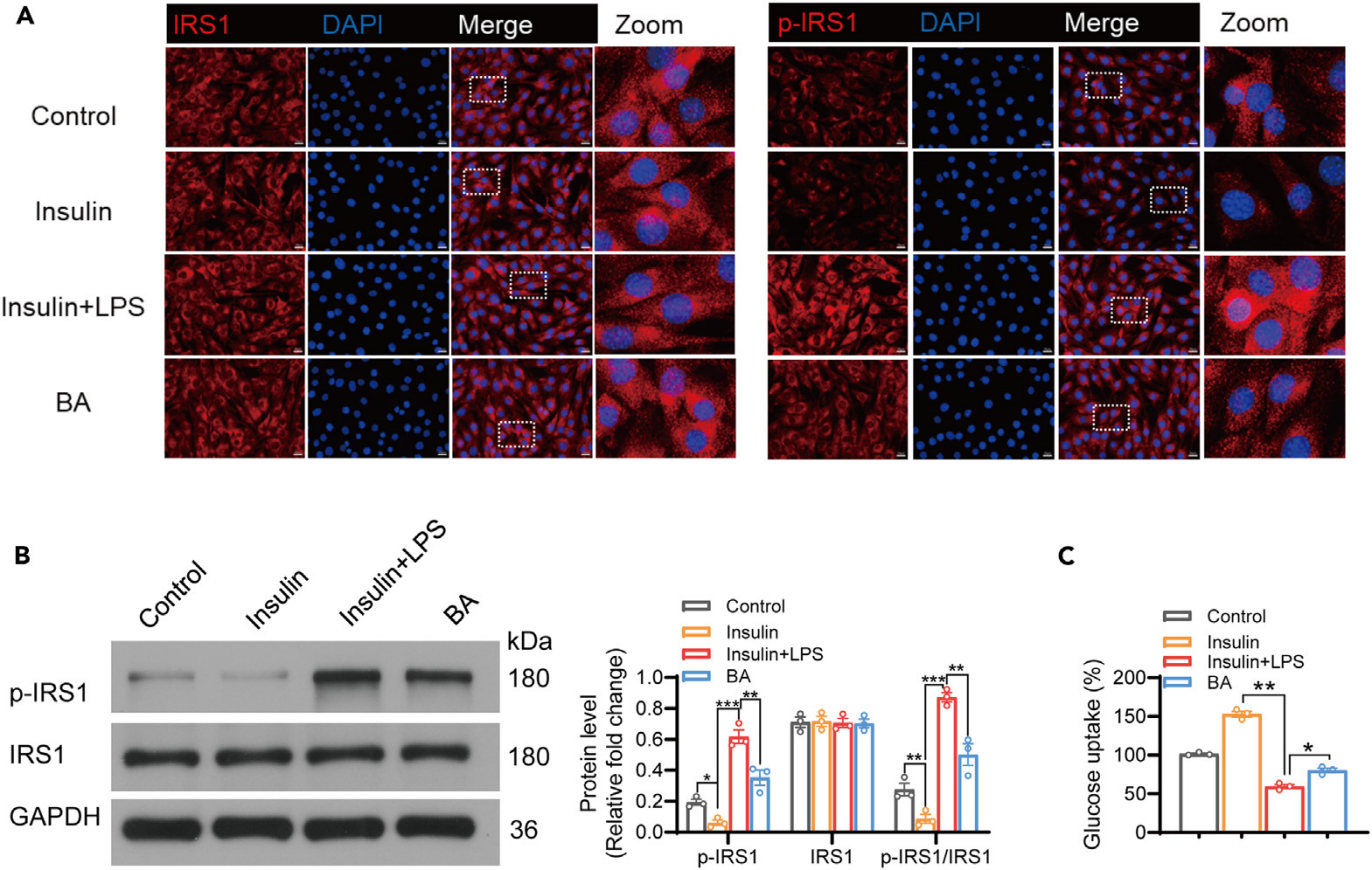
BA restores skeletal muscle glucose uptake to maintain insulin sensitivity (A and B) Mouse calf gastrocnemius muscle cells were treated with insulin, insulin + LPS, or BA, and the expression of IRS1 and pIRS1 was detected by immunofluorescence staining (A) and western blotting (B), respectively. The specific phosphor IRS site was S307. Scale bar, 20 μm; zoom is a 4x enlargement of the original image. (C) The level of glucose uptake in the four groups of skeletal muscle cells was evaluated using the fluorescent glucose analog 2-NBDG. Data represented as means ± SEM. *n* = 3 per group. One-way ANOVA. ∗*p* < 0.05, ∗∗*p* < 0.01 and ∗∗∗*p* < 0.001.

In patients with GDM, impaired skeletal muscle glucose uptake due to defects in insulin signal transduction leads to maternal hyperglycemia. Additionally, the production of TNF-α stimulated by LPS can induce similar impairments in skeletal muscles. We aimed to determine whether BA increases glucose uptake in skeletal muscle cells. To this end, we treated skeletal muscle cells with LPS, insulin, and BA. We found that LPS stimulated the phosphorylation of insulin receptor substrate 1 (IRS1), whereas the expression of pIRS1 significantly decreased after treatment with BA. The expression of pIRS1 in insulin-treated skeletal muscle cells was lower than in the control group (Figures 5A and 5B). Furthermore, the fluorescent glucose analog 2-NBDG was used to evaluate the glucose uptake capacity of skeletal muscle cells. Insulin increased glucose uptake in skeletal muscle cells, while LPS significantly inhibited this process. Interestingly, after treatment with BA, there was partial restoration of glucose uptake capacity in these skeletal muscle cells (Figure 5C). These results indicated that BA can restore glucose uptake in skeletal muscle cells.

### BA promotes the proliferation of islet β cells and restores their function

**Figure 6:**
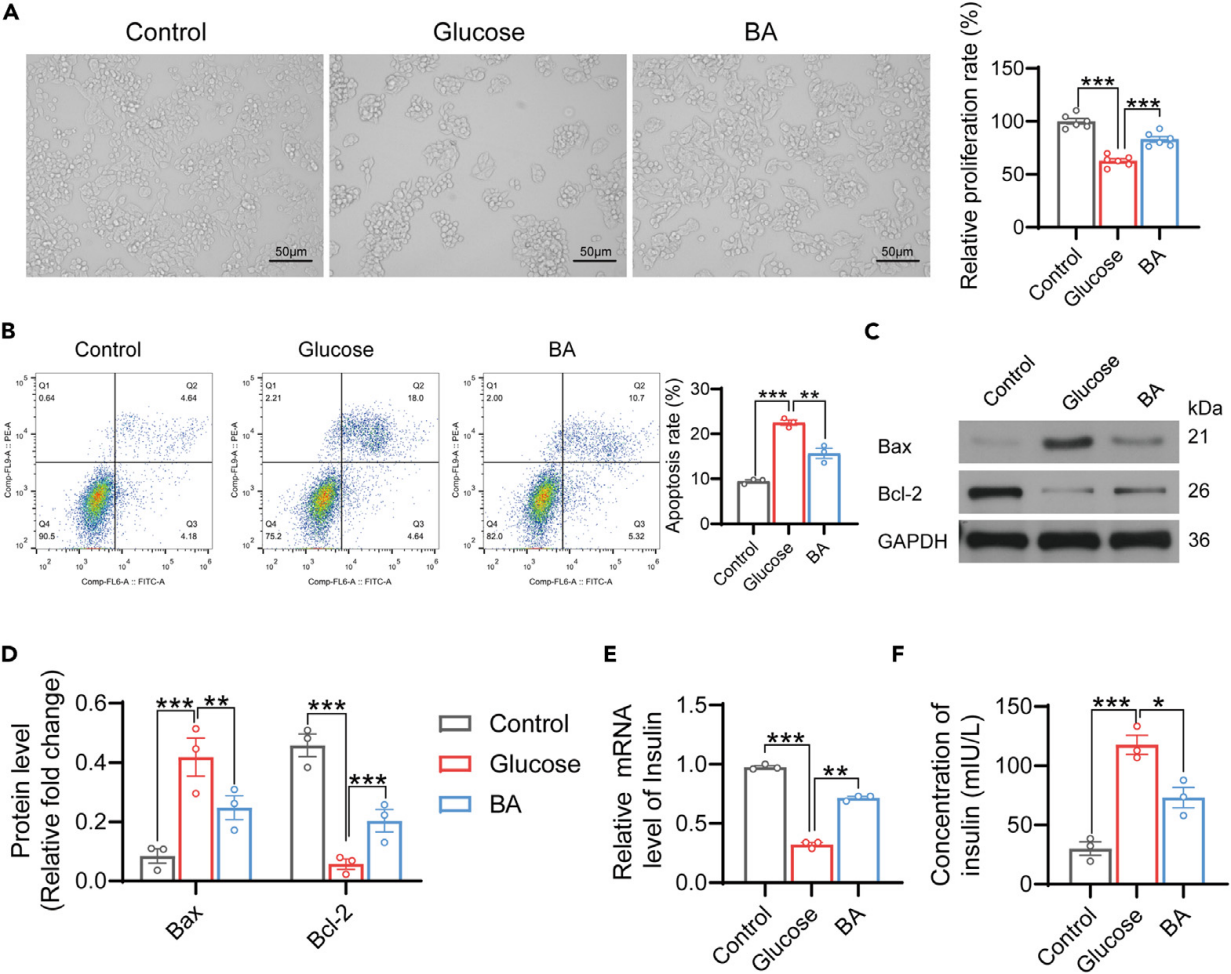
BA promotes the proliferation of islet β cells and restores their function (A) Mouse islet β cell line Min6 was divided into three groups: control, glucose and BA groups. The cell density observed under a microscope was used to estimate the proliferation of islet β cells. MTT assay was performed to evaluate the proliferation of islet β cells. *n* = 6. (B) Apoptosis of islet β cells in the three groups was assessed by flow cytometry. Apoptosis rate was shown on the right. *n* = 3. (C and D) The expression of apoptosis-related proteins Bax and Bcl-2 was detected by western blotting (C) and gray scale analysis. *n* = 3. (E and F) Relative mRNA expression of insulin (E) and the concentration of insulin in the medium (F) were determined by RT-qPCR and ELISA, respectively. *n* = 3. Data represented as means ± SEM. One-way ANOVA. ∗*p* < 0.05, ∗∗*p* < 0.01 and ∗∗∗*p* < 0.001.

To further investigate the impact of BA on islet β-cell function, we isolated and cultured these cells *in vitro* in a high-glucose medium, followed by treatment with BA. It was demonstrated that high glucose significantly reduced the density and proliferation of islet β cells, which could be effectively reversed by BA treatment (Figure 6A). Additionally, high glucose promoted the apoptosis of islet β cells, and the expression of Bax was elevated and that of Bcl-2 was decreased; However, BA treatment markedly inhibited apoptosis and reversed the expression of Bax and Bcl-2 (Figures 6B–6D). Furthermore, high glucose prominently inhibited the mRNA level and secretion of insulin in islet β cells, which was substantially restored by BA treatment (Figures 6E and 6F). These results suggest that BA has the potential to promote proliferation and restore islet β cell function.

### BA reduces inflammation and insulin resistance by inhibiting TLR4/NF-κB signaling pathway

**Figure 7:**
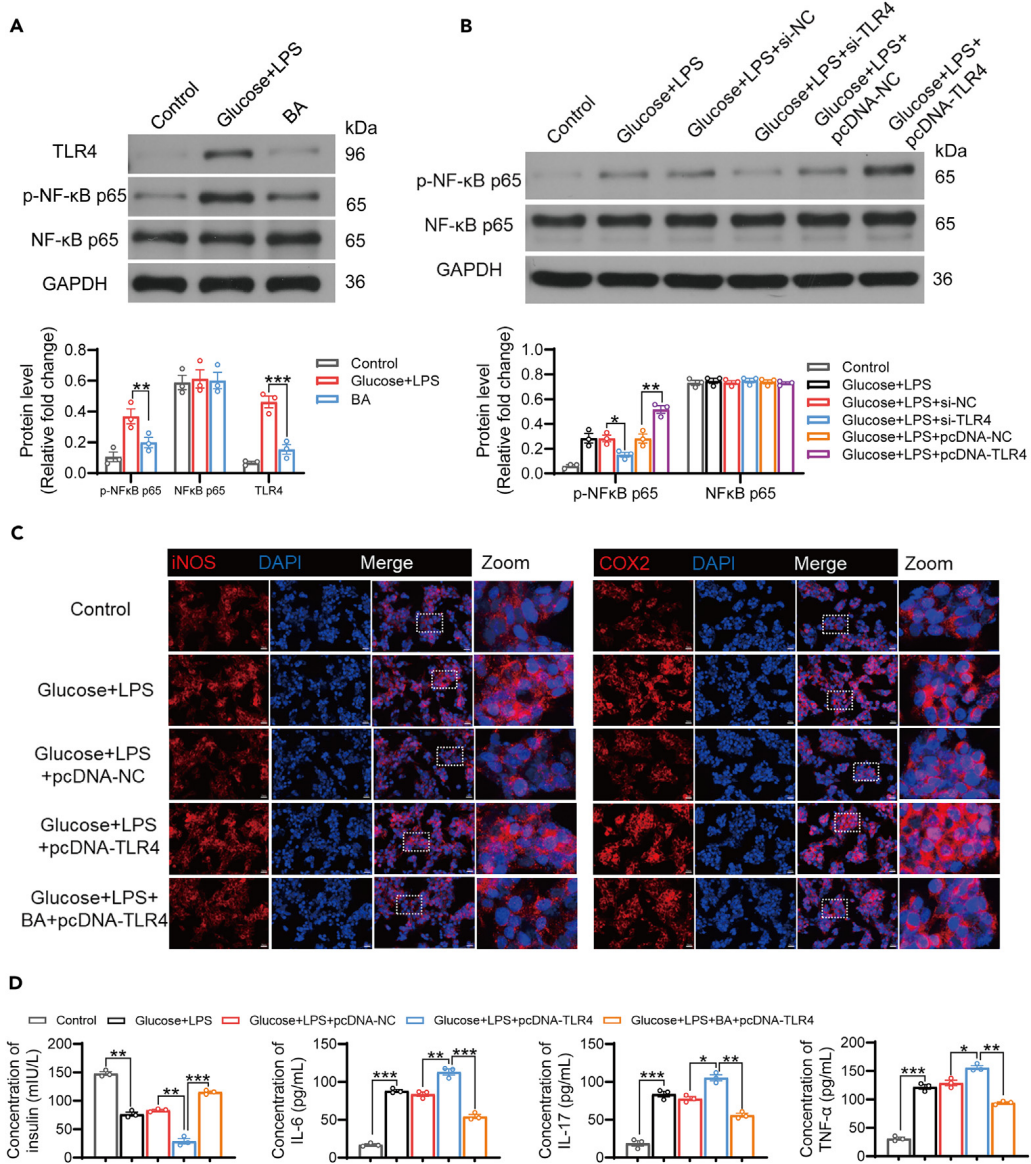
BA reduces inflammation and insulin resistance by inhibiting TLR4/NF-κB signaling pathway (A) Activation of the TLR4/NF-κB signaling pathway in the islet β cell line Min6 of the control, glucose plus LPS, and BA groups was verified by western blot assay. (B) The regulatory effect of TLR4 on NF-κB activation was demonstrated using western blotting. (C) The expression of two inflammation-related proteins, iNOS and COX2, was examined using immunofluorescence staining. Scale bar, 20 μm and zoom is a 4x enlargement of the original image. (D) The concentrations of insulin, IL-6, IL-17, and TNF-α in the medium of these five groups of islet β cells were tested by ELISA. Data represented as means ± SEM. *n* = 3 per group. One-way ANOVA. ∗*p* < 0.05, ∗∗*p* < 0.01 and ∗∗∗*p* < 0.001. See also Figures S3 and S4.

Although the inhibitory effects of BA on inflammation and IR have been observed, the specific regulatory mechanisms remain elusive. Previous studies have shown that activation of the TLR4/NF-κB signaling pathway is related to the pathogenesis of IR and diabetes.^27^^,^^28^ Moreover, we demonstrated activation of the TLR4/NF-κB/NLRP3 pathway in GDM mice, and BA notably inhibited the activation of this signaling pathway (Figures S3A–S3E). These findings suggest that BA effectively inhibits the inflammatory response in GDM mice, potentially by suppressing the TLR4/NF-κB/NLRP3 pathway, which BA regulate β-cell inflammation by modulating this pathway. To address this issue, we cultured islet β cells in high-glucose medium, followed by LPS stimulation and BA treatment. Our findings revealed that both glucose and LPS stimulation led to upregulation of TLR4 expression and activation of NF-κB, which was effectively reversed by BA (Figure 7A). Activation of NF-κB was regulated by TLR4, as evidenced by alterations in phosphorylated NF-κB p65 expression following transfection with pcDNA-TLR4 and si-TLR4. Transfection with pcDNA-TLR4 in combination with glucose and LPS stimulation facilitated the activation of NF-κB, whereas transfection with si-TLR4 inhibited the activation of NF-κB (Figures 7B and S4A). Furthermore, the expression of inflammation-related proteins iNOS and COX2 was highest in glucose+LPS+pcDNA-TLR4 group, while significantly inhibited by BA treatment (Figures 7C and S4B). Additionally, we observed an upregulation in the secretion of the proinflammatory cytokines IL-6, IL-17, and TNF-α following treatment with glucose and LPS. Notably, this trend was further augmented upon transfection with pcDNA-TLR4; however, this was reversed by BA treatment. Moreover, glucose and LPS stimulation significantly inhibited insulin secretion by islet β cells, which was further inhibited after transfection with pcDNA-TLR4, but markedly reduced following BA treatment (Figure 7D). Conversely, knockdown of TLR4 in cells prior to BA treatment no longer changed the levels of these inflammatory markers in supernatants (Figure S4C). These findings suggest that BA mitigates inflammation and IR via inhibition of the TLR4/NF-κB signaling pathway.

## Discussion

GDM predominantly manifests during the perinatal period in pregnant women, and its incidence has been progressively increasing over time. As the disease progresses, it can lead to hypertension, reproductive system disorders, excessive weight gain, fetal malformation, respiratory distress, and even neonatal mortality, posing a significant threat to both maternal and infant health.^30^ Currently, the main treatment of GDM primarily involves hypoglycemic agents and insulin injections, yet their impact on maternal and infant remains suboptimal.^3^ In recent years, dietary interventions for GDM have garnered significant attention. Several studies have reported the therapeutic potential of BA in T2DM; however, its role in GDM has not yet been investigated. In this study, we demonstrated that administration of BA at a dosage of 10 mg/mL daily during pregnancy maintained glucose homeostasis in GDM mice and improved birth outcomes in offspring. These findings suggested that BA is a promising natural fatty acid for the treatment and amelioration of GDM.

Islet β-cell dysfunction and IR are two crucial factors in the pathogenesis of GDM.^31^ During pregnancy, the body’s demand for insulin increases, necessitating enhanced β-cell function.^32^ A large number of scholars believe that the decrease and dysfunction of islet β cells are closely related to the occurrence and development of GDM.^33^^,^^34^^,^^35^ Our study showed that BA treatment restored islet morphology and increased β-cell number, thereby improving insulin secretion under glucose stimulation by inhibiting the TLR4/NF-κB signaling pathway. The activation of inflammatory pathways precipitates the creation of an inflammatory microenvironment within islets, consequently compromising the insulin secretory capacity of β cells and precipitating IR.^22^ Studies have shown that suppression of the TLR4/NF-κB signaling cascade can mitigate islet inflammation, ameliorate β-cell functionality, and restore glucose homeostasis.^36^ A study demonstrated that a diet rich in BA resulted in decreased postprandial inflammation.^37^ Utilizing an advanced structure-based computational strategy coupled with molecular dynamics simulations, Nath et al. reported that BA exhibits a significant affinity for FFA1R, potentially functioning as an agonist.^20^ This interaction may augment the insulin secretion. The potential of BA to stimulate insulin release via the FFA1R pathway remains an intriguing avenue for future research.

The skeletal muscle, the main target tissue of glucose oxidation and insulin action, is the target organ of IR. The insulin-dependent signaling pathway is the main mechanism that increases glucose transport in skeletal muscle.^38^ When the insulin signaling pathway is impaired, the uptake and utilization of glucose in skeletal muscle is impaired, leading to IR.^38^ IRS1 is a key molecule that regulates the insulin-signaling pathway. Under physiological conditions, insulin binds to the insulin receptor on skeletal muscle cells to activate IRS1, which initiates downstream signal transduction pathways.^39^ Studies have shown that the abnormal expression level of IRS1 and its serine phosphorylation can lead to disruption of insulin signal transduction, thereby inducing IR.^40^ In this study, we stimulated skeletal muscle cells with LPS and found that BA significantly reduced p-IRS1 (Ser307) expression. Consistent with our findings, Li et al. showed that liraglutide ameliorated palmitate-induced IR by inhibiting the serine phosphorylation of IRS-1 in mouse skeletal muscle cells.^41^ In addition, BA restored glucose uptake in skeletal muscle cells. These findings suggest that BA can improve IR.

Similar to other types of diabetes, the role of inflammatory factors in GDM occurrence and progression cannot be ignored. In addition to hormone levels, the most significant change that occurs in pregnant women before and during pregnancy is the chronic inflammatory response that occurs in the placenta during pregnancy.^42^ Recent mechanistic investigations propose a link between GDM and a state of persistent, low-grade inflammation, often referred to as “meta-inflammation”, which is distinct from the typical acute inflammatory response.^43^ This form of inflammation is not attributed to bacterial or mycoplasma infections but is instead triggered by the presence of aberrant metabolites, such as LPS, short-chain fatty acids, and branched-chain fatty acids.^42^ LPS is the main component of the outer membrane of gram-negative bacteria. Ligated TLR4 then transduces the signal to activate NF-κB-mediated transcription of genes encoding molecules of the immune system, including cytokines and chemokines.^23^ Our research has identified an upregulation of pro-inflammatory cytokines such as IL-6, IL-17, and TNF-α, along with an increased infiltration of CD68-positive macrophages in the pancreas and liver of GDM mice. These findings underscore the presence of a pronounced inflammatory response in GDM, corroborating the work of Jiao et al.^44^^,^^45^ At the cellular level, we replicated the inflammatory conditions of GDM by culturing islet β cells in a high-glucose medium and exposing them to LPS. The LPS-induced inflammation model, serves as a valuable tool for *in vitro* studies of GDM, providing a controlled environment to investigate the disease’s inflammatory mechanisms and potential therapeutic interventions. This experimental setup mimics the inflammatory milieu observed in GDM, and our results indicate that the treatment with BA mitigates the inflammatory response by suppressing the TLR4/NF-κB pathway.

In conclusion, BA was found to reduce glucose levels, improve glucose tolerance and insulin sensitivity, and enhance birth outcomes in GDM offspring. Further studies showed that BA mitigated inflammation and IR in GDM by inhibiting the TLR4/NF-κB signaling pathway. Our findings provide a basis for the clinical application of BA in GDM treatment.

### Limitations of the study

In this study, we found that BA improves pancreatic β-cell function by inhibiting the TLR4/NF-κB signaling pathway; however, BA has demonstrated a robust affinity for FFA1R, suggesting its potential to act as an agonist. This interaction could potentially facilitate insulin secretion, presenting an engaging frontier for future research. A dose of 10 mg/mL BA was meticulously selected for the treatment of GDM in our murine model. Given the current findings and considering the paucity of reference data, we are constrained from asserting that the dosage administered to the animal model corresponds to physiological levels. The therapeutic efficacy of BA in GDM requires further validation through extensive clinical studies. In addition to abnormal glucose metabolism and inflammation, abnormal lipid metabolism and oxidative stress also occur in GDM. The ability of BA to ameliorate these additional pathological aspects of GDM warrants further in-depth investigation.

## Resource availability

### Lead contact

Further information and requests for resources and reagents should be directed to and will be fulfilled by the Lead Contact, Minkai,Cao (Caominkai2008@163.com).

### Materials availability

This study did not generate new unique reagents.

### Data and code availability

• All data reported in this paper will be shared by the lead contact upon request.
• This paper does not report original code.
• Any additional information required to reanalyze the data reported in this paper is available from the lead contact upon request.

## Acknowledgments

This work was supported by the 10.13039/501100001809National Natural Science Foundation of China (grant no. 82401994), the Top Medical Expert Team of the Wuxi Taihu Talent Plan (grant no. DJTD202106, GDTD202105), Medical Key Discipline Program of Wuxi Health Commission (grant no. ZDXK2021007, CXTD2021005); Top Talent Support Program for Young and Middle-aged People of Wuxi Health Committee (grant no. BJ2023090, HB2023079), Scientific Research Program of Wuxi Health Commission (grant no. Z202109, M202208, and M202217), Wuxi Science and Technology Development Fund (grant no. N20202003, Y20222001), Wuxi Maternal and Child Health Research Project (grant no. FYKY202103). Thanks to BioRender software (http://biorender.com), the graphical abstract was created with BioRender.com with permission.

## Author contributions

K-R.L., Y.G., and X-N.P.: investigation, methodology, writing—original draft. S.C.: methodology and visualization. L.Z. and C.J.: conceptualization, project administration, funding acquisition, data curation, supervision, writing, review, and editing. M-K.C.: Investigation, methodology, writing the original draft, visualization, and formal analysis.

## Declaration of interests

The authors declare no conflicts of interest.

## STAR★Methods

### Key resources table

**Table:** 

REAGENT or RESOURCE	SOURCE	IDENTIFIER
**Antibodies**		

Rabbit anti-Insulin	Abcam	Cat#ab133281; RRID: AB_11157959
Rabbit anti- CD68	Abcam	Cat#ab283654; RRID: AB_2922954
Donkey anti-rabbit IgG	Abcam	Cat#ab205722; RRID: AB_2904602
Rabbit anti-insulin	Abcam	Cat#ab181547; RRID: AB_2716761
Mouse anti-Ki67	Abcam	Cat#ab279653; RRID: AB_2934165
Rabbit anti-iNOS	Abcam	Cat#ab178945; RRID: AB_2861417
Rabbit anti-IRS1 (phosphor S307)	Abcam	Cat#ab5599; RRID: AB_304975
Mouse anti-TLR4	Abcam	Cat#ab22048; RRID: AB_446735
Rabbit anti-NLRP3	Abcam	Cat#ab314905; RRID: N/A
Rabbit anti-NF-κB p65	Abcam	Cat#ab32536; RRID: AB_776751
Rabbit anti-NF-κB p65 (phospho T254)	Abcam	Cat#ab131100; RRID: AB_11157293
Rabbit anti-IRS1	Abcam	Cat#ab131487; RRID: AB_11156361
Rabbit anti-COX2	Abcam	Cat#ab179800; RRID: AB_2894871
Donkey anti-rabbit	Abcam	Cat#ab150075; RRID: AB_2752244
Goat anti-mouse	Abcam	Cat#ab150113; RRID: AB_2576208
Mouse anti-GAPDH	Abcam	Cat#ab8245; RRID: AB_2107448

**Chemicals, peptides, and recombinant proteins**		

STZ	Sigma, US	Cat#V900890
BA	Selleck, China	Cat#S5381
Insulin	Beyotime, China	Cat#P3376
Glucose	Sigma, US	Cat#G7021
LPS	Sigma, US	Cat#L4291
2-NBDG	Thermo, US	Cat#N13195
PI solution	Solarbio, China	Cat#P8080
DMSO	Sinopharm Chemical Reagent Co, China	Cat#30072418
Paraformaldehyde	Sinopharm Chemical Reagent Co, China	Cat#80096618
Hematoxylin	Sigma, US	Cat#H9627
DAPI	Sigma, US	Cat#D8417
BSA	Biofroxx, China	Cat#4240GR250
Collagenase Type I	Icell, China	Cat#iCell-17100-017
H_2_O_2_	Sinopharm Chemical Reagent Co, China	Cat#10011208
donkey serum		

**Critical commercial assays**		

Mouse ADP (Adiponectin) ELISA Kit	ELK Biotechnology, China	Cat#ELK1234
Mouse Insulin (INS) ELISA Kit	Cusabio, China	Cat#CSB-E05071m
Mouse IL6 (Interleukin 6) ELISA Kit	ELK Biotechnology, China	Cat#ELK1157
Mouse IL17 (Interleukin 17) ELISA Kit	ELK Biotechnology, China	Cat#ELK1147
Mouse GROβ (Growth Regulated Oncogene Beta) ELISA Kit	ELK Biotechnology, China	Cat#ELK1383
Mouse MCP2 (Monocyte Chemotactic Protein 2 ELISA Kit	ELK Biotechnology, China	Cat#ELK8888
Mouse MIP1α (Macrophage Inflammatory Protein 1 Alpha) ELISA Kit	ELK Biotechnology, China	Cat#ELK1116
Mouse endotoxin (ET) ELISA Kit	Mlbio, China	Cat#ml002005
Mouse TNFα (Tumor Necrosis Factor Alpha) ELISA Kit	ELK Biotechnology, China	Cat#ELK1387
HRP/DAB Detection Kit	ZSGB-Bio, China	Cat#ZLI-9019
TRIpure Total RNA Extraction Reagent	ELK Biotechnology, China	Cat#EP013
EntiLink™ 1st Strand cDNA Synthesis Super Mix	ELK Biotechnology, China	Cat#EQ031
BCA Kit	ASPEN, China	Cat#AS1086
ECL developer	ASPEN, China	Cat#AS1012
EnTurbo™ SYBR Green PCR SuperMix	ELK Biotechnology, China	Cat#EQ001
Cell Counting Kit-8	Beyotime, China	Cat#C0038

**Experimental models: cell lines**		

Min6	Shanghai Cell Bank	N/A
Placenta tissue explants	This paper	N/A
Mouse skeletal muscle cells	This paper	N/A

**Experimental models: organisms/strains**		

C57BL/6J mice	Beijing Vital River Laboratory Animal Technology Co., LTD	IMSR Cat# JAX:000664;

**Oligonucleotides**		

si-NC	Ribobio, China	N/A
si-TLR4	Ribobio, China	N/A
See Table S1 for Primers	N/A	N/A

**Recombinant DNA**		

pcDNA-NC	This paper	N/A
pcDNA-TLR4	This paper	N/A

**Software and algorithms**		

ImageJ	NIH	https://imagej.nih.gov/ij/
Prism	Graphpad	https://www.graphpad.com

**Other**		

Normal chow diet	Research Diets	D12450J
High fat diet	Wajx bio-technology, China	D12492

### Experimental model and study participant details

#### Animal studies

SPF C57BL/6J mice aged 6–8 weeks were obtained from Beijing Vital River Laboratory Animal Technology Co., Ltd. A total of 60 female and 30 male mice were housed in the SPF animal room of the Laboratory Animal Center of JiangNan University (temperature: 22°C–26°C, light-dark cycle: 12 h each). Male and female mice were housed in cages with free access to water and diet, and the bedding material was changed regularly. This study has been reviewed and approved by the Ethics Committee children’s Hospital of Wuxi (Approval WXCH2021-09-012).

After one week of adaptive feeding, the female mice were randomly assigned to three groups: normal control group (Control), GDM Model group (Model) and BA treatment group (BA). The mice in the control group were fed a normal diet, whereas those in the Model and BA groups were fed a HFD. After 4 weeks of feeding, the female and male mice were mated at a ratio of 2:1 for 3 days. Pregnant female mice were included in this experiment, and the day on which the vaginal plug was observed in the vagina of female mice was counted as the first day of gestation (GD1). Subsequently, the pregnant mice in the Model and BA groups were intraperitoneally injected with fresh STZ solution (30 mg/kg) once every 24 h for a total of three times, and the control group was injected with an equivalent amount of citrate buffer (0.1 mol/L, pH = 4). The model was successfully established when the random blood glucose level of the pregnant mice was higher than 11.1 mmol/L. Thereafter, the BA group was intraperitoneally injected with BA (10 mg/kg) daily, and the Control group and the Model group were given the same volume of normal saline until GD17.

After delivery (between weaning and 3 weeks of age), the body weight of the surviving pups and the number of dead pups were recorded, and glycometabolism indexes were determined. For sex identification of neonatal mice, the initial basis is the length of the distance between the genitals and anus is the projection of the genitals. The sex-determining region Y (Sry) has been identified in the muse Y chromosomes.^46^ Primers were designed for the Sry gene, and PCR was performed using neonatal mouse genomic DNA. After agarose horizontal gel electrophoresis, male mice showed a stripe of 282 bp, whereas female mice did not. Fasting blood glucose (FBG), oral glucose tolerance test (OGTT), and intraperitoneal insulin tolerance test (IPITT) were performed on the offspring of mice at 3 weeks of age (i.e., after weaning) in the same manner as in adult mice.

### Method details

#### Cell culture

Mouse islet β cell line Min6 was obtained from Shanghai Cell Bank, Chinese Academy of Sciences. Cells were cultured in DMEM (Hyclone, US) supplemented with 10% fetal bovine serum (Gibco, US) and 1% dual-antibodies (Hyclone, US). The cells were cultured at 37°C in an incubator with 5% CO_2_.

To explore the effect of BA on islet β-cell function, Min6 cells were cultured in medium containing high glucose for 24 h and then treated with BA. To explore the effect of BA on the TLR4/NF-κB pathway, Min6 cells were cultured in high-glucose medium, stimulated with 10 μg/mL LPS for 1 h, and then treated with BA (2 μM) for 24 h.

#### Metabolic characteristics

For glucose metabolism analysis, mice were fasted for 14–16 h, and then subjected to FBG levels or OGTT (1 g/kg body weight) were performed. IPITT (1 unit of insulin/kg body weight, i.p.) was administered. Mice were fasted for 4–6 h for IPITT. Blood samples were collected from the tail vein and the blood glucose level was measured by the glucose meter (Roche, Swiss). A blood glucose curve was drawn, and the area under the curve (AUC) was calculated.

#### Enzyme-linked immunosorbent assay (ELISA)

The Insulin Assay Kit (Njjcbio, China) and Adiponectin Assay Kit (Njjcbio, China) were used to quantify insulin and adiponectin (ADP) levels, respectively. This procedure was performed according to the manufacturer’s instructions. Briefly, gradient concentrations of the standard and the sample to be tested were added to a 96-well plate, followed by the addition of horseradish peroxidase-labeled antibody and allowed to react at 37°C for 1 h. After thorough washing, color-developing solutions A and B were added and incubated in the dark for 15 min. The absorbance at 450 nm was measured immediately after the addition of the stop solution. Serum insulin and ADP concentrations were determined using a standard curve.

In addition, the levels of pro-inflammatory cytokines IL-6, IL-17, and TNF-α, and chemokines CCL3, CCL8, CXCL2, and CXCL4 in the cell culture supernatant were quantified using ELISA kits purchased from Liankebio (China). The experimental procedure was the same as that described above.

#### Homeostasis model assessment of insulin resistance (HOMA-IR)

HOMA-IR was determined according to the following formula, based on the detected fasting glucose and insulin concentrations:

HOMA-IR = fasting plasma glucose (mmol/L) × fasting insulin (mU/L)/22.5.

#### Collection and processing of specimens

The mice were anesthetized and sacrificed by cervical dislocation, followed by rapid thoracotomy to remove intact pancreas and liver. One part was fixed in 4% formaldehyde solution, and the other part was frozen in a −80°C refrigerator. Simultaneously, the placenta was removed and one thigh was cut from the hip bone of the mice after removing the skin, fascia, and adipose tissue. The calf gastrocnemius muscle of the mice was completely removed and stored at −80°C.

Tissue explants were used to measure the levels of proinflammatory cytokines and chemokines in the placenta. Briefly, the tissues were washed with PBS buffer to remove connective tissue and blood vessels, placed in DMEM containing 1% penicillin-streptomycin, and incubated at 37°C for 1 h. After pre-treatment with BA for 1 h, 10 μg/mL LPS was added, and incubation was continued for 24 h.

The mouse calf gastrocnemius muscle was used to examine glucose uptake by skeletal muscle cells. Skeletal muscles were chopped after the removal of fat and connective tissue, washed with PBS, and digested in DMEM containing 0.2% type I collagenase for 1 h. The digested liquid was filtered through a 70 μm cell filter and centrifuged at 500×g for 10 min. After discarding the supernatant, the cells were resuspended in 5 mL of DMEM/F12 medium and cultured in a cell incubator.

#### Histological analysis

Pancreas and liver tissues from the same areas of mice were collected, immersed in formalin solution (Macklin, China), and fixed overnight. Following dehydration in gradient ethanol, transparency in xylene, and embedding in paraffin, 4 μm thick tissue sections were prepared. For histological analysis, the sections were dewaxed and rehydrated before staining.

For H&E staining, sections were sequentially treated with hematoxylin and eosin (Phygene, China). Subsequently, excess staining solution was washed away before observation under an optical microscope.

The immunohistochemical (IHC) staining kit was purchased from ZSGB-Bio (China). The sections were initially subjected to antigen retrieval and then immersed in 3% H_2_O_2_ -methanol solution for 10 min to eliminate endogenous catalase. Subsequently, sections were blocked with 10% donkey serum at room temperature for 30 min, followed by incubation with appropriately diluted primary antibodies. After incubation at 37°C for 1 h and rinsing with PBS, the sections were exposed to secondary antibodies and further incubated at 37°C for another hour. DAB color-developing solution was added, and the reaction time was carefully monitored under a microscope. The sections were counterstained with hematoxylin for 1 min. After washing, sections were dehydrated, transparent, and sealed. The results were observed and analyzed under a microscope. The primary antibodies used were anti-insulin (ab133281, 1:300 dilution; Abcam) and anti-CD68 (ab283654, 1:100 dilution; Abcam). Donkey anti-rabbit IgG (ab205722, 1:500 dilution, Abcam) was used as secondary antibody.

#### Immunofluorescence staining

Pretreated tissue sections and fixed cells were incubated in a humidified box with diluted primary antibodies, including anti-insulin (ab181547, 1:200 dilution, Abcam), anti-Ki67 (ab279653,1:50 dilution, Abcam), anti-iNOS (ab178945, 1:500 dilution, Abcam), anti-COX2 (ab179800, 1:200 dilution, Abcam), anti-IRS1 (ab131487, 1:100 dilution, Abcam), anti-IRS1 (phosphor S307) (ab5599, 1:100 dilution, Abcam), anti-TLR4 (ab22048, 1:100 dilution, Abcam), anti-NLRP3 (ab314905, 1:100 dilution, Abcam), anti-NF-κB p65 (ab32536, 1:100, Abcam), anti-NF-κB p65 (phospho T254) (ab131100, 1:100 dilution, Abcam), and anti-GAPDH (ab8245, 1:1000, Abcam). After incubation for 1 h at room temperature, the sections were washed 3 times with PBS and subsequently treated with secondary antibody solutions, including donkey anti-rabbit (ab150075, 1:500 dilution, Abcam) and goat anti-mouse (ab150113, 1:500 dilution, Abcam). After another hour of incubation at room temperature, the sections were washed and sealed for observation and imaging under a fluorescence microscope.

#### RT-qPCR

First, total RNA was extracted from tissues or cells using a MolPure TRIeasy Plus Total RNA Kit (Yeasen, China), and the concentration and purity of RNA were assessed. Subsequently, reverse transcription was performed using the HiScript 1st Strand cDNA Synthesis Kit (Vazyme, China), with the extracted RNA as a template. Next, the specific primer mixture for the target genes was mixed with SYBR dye (Roche, Swiss) and cDNA in a 96-well plate for PCR amplification. The results were analyzed using software for relative quantification. Primer sequences used were listed in Table S1.

#### Endotoxin detection

Plasma endotoxin levels in mice were quantified using a dynamic chromogenic limulus test kit (Solarbio, China). In brief, 100 μL of water for the bacterial endotoxin test, endotoxin standard solution, and samples were added to the heat removal microplate. The plate was then placed in a Limulus test microbial rapid detection system (ELx808IU-SN, Bio-tek, US) and preheated at 37°C for 10 min. Subsequently, 100 μL of Tachypleus amebocyte lysate was added to each well, quickly mixed, and incubated at 37°C for 2 h. OD values were measured every 30 s at a wavelength of 405 nm. A standard curve was constructed to determine the concentration of endotoxins in the samples.

#### Western blot

Skeletal muscle cells were collected and incubated with pre-cooled RIPA lysate (Bioss, China) at 4°C for 30 min, followed by centrifugation at 12,000 × g for 10 min at 4°C to obtain the supernatant. To ensure a consistent loading volume, the concentration of protein samples in each group was detected using a BCA kit (ThermoFisher, US). Subsequently, the samples were diluted with 5× bromophenol blue loading buffer to a protein concentration of 5 μg/μL and boiled for 10 min. SDS polyacrylamide gel electrophoresis was performed to separate the protein samples. After electrophoresis, the separated protein bands were electrotransferred onto a PVDF membrane (Millipore, US). The PVDF membrane was blocked in 5% skim milk for 2 h at room temperature. Subsequently, the PVDF membranes were incubated with diluted primary antibodies at 4°C overnight. The next day, the membranes were washed three times with TBST buffer and incubated with the secondary antibody solution for 1 h. After washing again, the PVDF membrane was placed in a chemiluminescence imager (Bio-Rad, ChemiDoc MP, US), and the protein bands were observed and analyzed by dripping an ECL developer (Beyotime, China) on the membrane. The following antibodies were used: anti-IRS1 (ab131487, 1:100 dilution, Abcam), anti-IRS1 (phospho S307) (ab5599, 1:100 dilution, Abcam), anti-NF-κB p65 (ab32536, 1:5000, Abcam), anti-NF-κB p65 (phospho S536) (ab76302, 1:1000 dilution, Abcam), anti-Bax (ab182733, 1:2000, Abcam), anti-Bcl-2 (ab182858, 1:2000, Abcam), anti-GAPDH (ab9485, 1:1000, Abcam), and goat anti-rabbit IgG H&L (ab182016, 1:20000, Abcam).

#### Glucose uptake test

2-NBDG, a D-glucose analog equipped with a fluorophore, was used to quantify glucose uptake in viable cells. Skeletal muscle cell cultures were supplemented with 100 μM 2-NBDG (Bidepharm, China) and incubated at 37°C for 30 min. The cells were then rinsed with PBS, observed, and photographed using a fluorescence microscope (Olympus BX53, Japan). The fluorescence intensity of the cells was determined using the ImageJ software.

#### Flow cytometry

Flow cytometry was performed to evaluate apoptosis. Islet β cells in control, glucose and BA group were collected (cell number: 1×10^6^ cells/mL) and centrifuged at 1000 rpm for 5 min. After washing with PBS, the cells were fixed with 70% ice-cold ethanol at 4°C for 1 h and then resuspended in PBS. The islet β-cells were filtrated through a 400 mesh sieve and centrifuged again at 1000 rpm for 5 min. The cells were then stained with PI solution (Sigma, US) for 30 min and analyzed using a flow cytometer (BD AccuriC6, US).

#### MTT assay

The islet β-cell line Min6 was seeded into 96-well plates, and the cells were treated according to the grouping. Subsequently, 10 μL of the MTT reagent (Acmec, China) was added to each well and incubated for 4-h incubation. After terminating the culture, the supernatant was carefully aspirated and replaced with 100 μL DMSO (Aladdin, China) to fully dissolve the formazan crystals. The plate was shaken for 10 min on a shaker for complete dissolution. Finally, the absorbance at 490 nm was measured using a microplate reader (BioTek Synergy2, US).

### Quantification and statistical analysis

*The in vitro* experiments were repeated at least three times, and the *in vivo* assays were repeated twice, with the number per condition included in the figure legends. Additional data were plotted and analyzed using Prism 8.0 software (GraphPad Software, San Diego, CA). Comparisons were performed using the Student’s t test between two groups or ANOVA for multiple groups. Two-way AVOVA with multiple comparisons were used for GTT and ITT studies. Results are presented as the mean ± SEM. *p* < 0.05 is considered statistically significant.
