# Supplementary material for: Behenic acid alleviates inflammation and insulin resistance in gestational diabetes mellitus by regulating TLR4/NF-κB signaling pathway
Source: iScience. 2024 Sep 24;27(10):111019. doi: 10.1016/j.isci.2024.111019 (PMC11490720; doi:10.1016/j.isci.2024.111019)
Supplement: Document S1. Figures S1–S4 and Table S1 [file mmc1.pdf]

iScience, Volume *27*

Supplemental information

Behenic acid alleviates inflammation and insulin resistance in gestational diabetes mellitus by regulating TLR4/NF-κB signaling pathway

Kerong Liu, Ying Gu, Xingnan Pan, Sha Chen, Jie Cheng, Le Zhang, and Minkai Cao

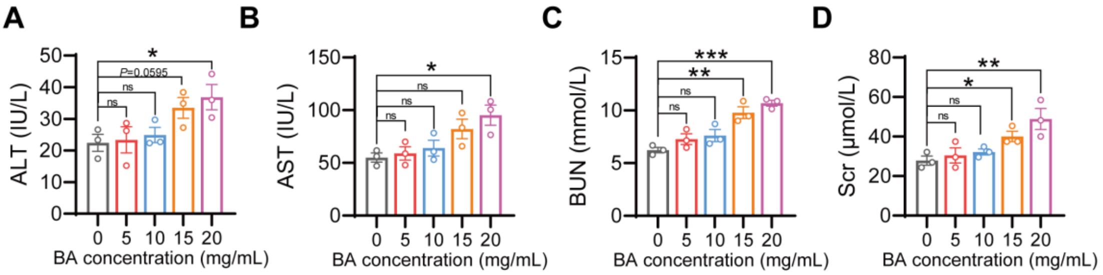

Figure S1. BA pre-experimental concentrations were determined. Related to Figure 1.

(A-D) After daily administration of 0, 5, 10, 15, and 20 mg/mL BA to female mice, serum alanine aminotransferase (ALT), aspartate aminotransferase (AST), blood urea nitrogen (BUN), and serum creatinine (Scr) levels were measured 17 days later. n=3. Data represented as means ± SEM. One-way ANOVA. *$P<0.05$, **$P<0.01$, ***$P<0.001$. ns, not significant.

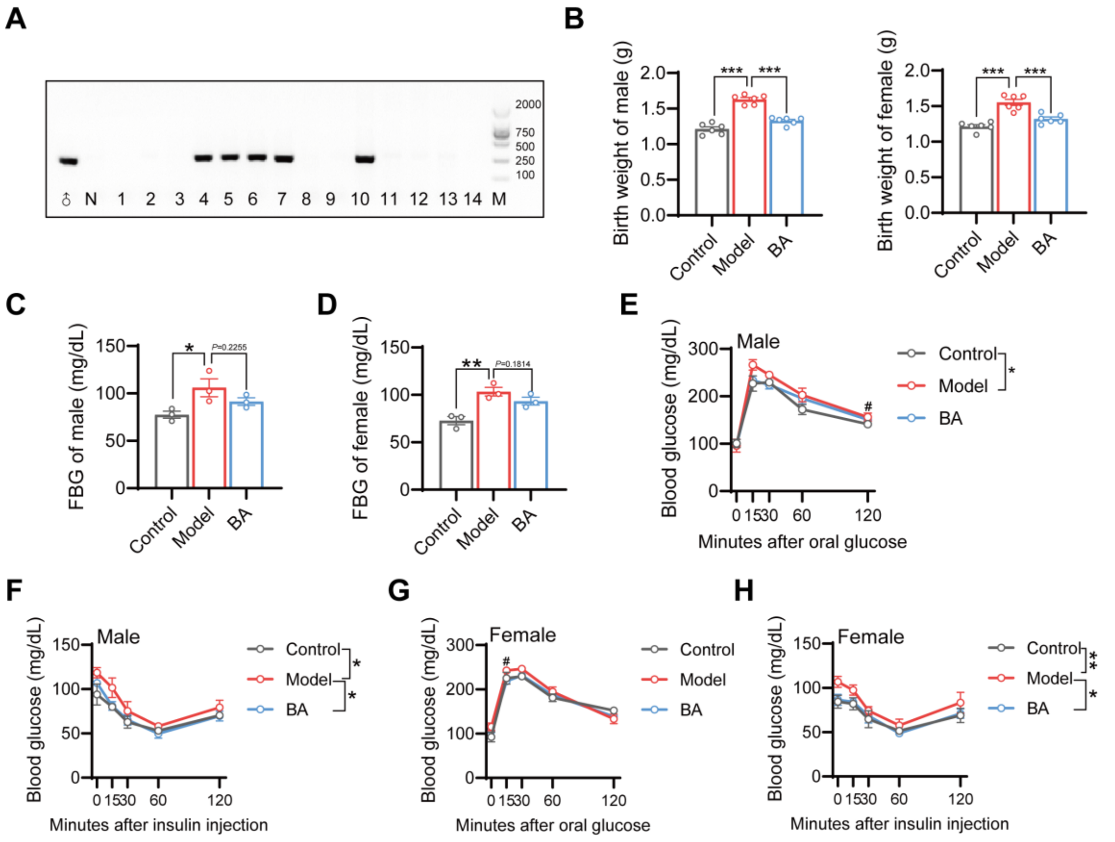

Figure S2. BA improves female and male birth outcomes in GDM mice offspring. Related to Figure 2.

(A) PCR analysis of genomic DNA from neonatal mice. Male mice showed a 282 bp stripe, whereas female mice did not. The first sample on the left was a positive reference for a known male. N, was used as the negative control.

(B) Birth weights of neonatal female and male mice were measured. n=6.

(C and D) Fasting blood glucose concentrations in male (C) and female (D) mice were measured at 3 weeks. n=3.

(E-H) Blood glucose curves of GTT (E) and ITT (F) in 3-week-old male mice, and GTT (G) and ITT (H) in female mice. n=3.

Data represented as means ± SEM. One-way ANOVA for B-D; Two-way ANOVA for E-H. *P<0.05, **P<0.01, ***P<0.001. $^{\#}P$<0.05 vs. Model/BA group.

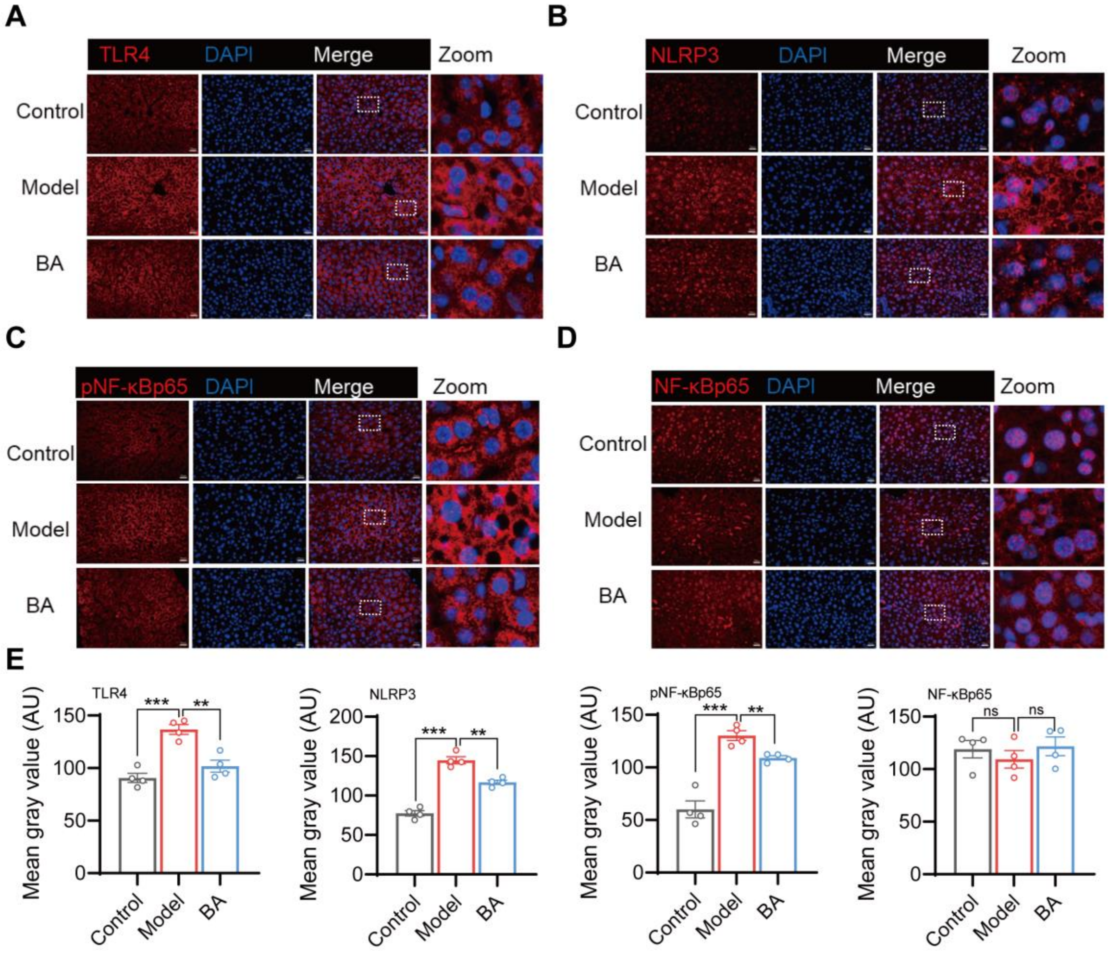

Figure S3. BA inhibits hepatic TLR4/NF-κB signaling pathway in GDM mice. Related to Figure 7.

(A-D) Activation of the TLR4/NF-κB/NLRP3 signaling pathway in the liver of mice was assessed by immunofluorescence staining. Scale bar = 20 μm; zoom is a 4x enlargement of the original image.

(E) Quantification of the mean fluorescence intensity. Data represented as means ± SEM. n=4. One-way ANOVA. ***P*<0.01, ****P*<0.001. ns, not significant.

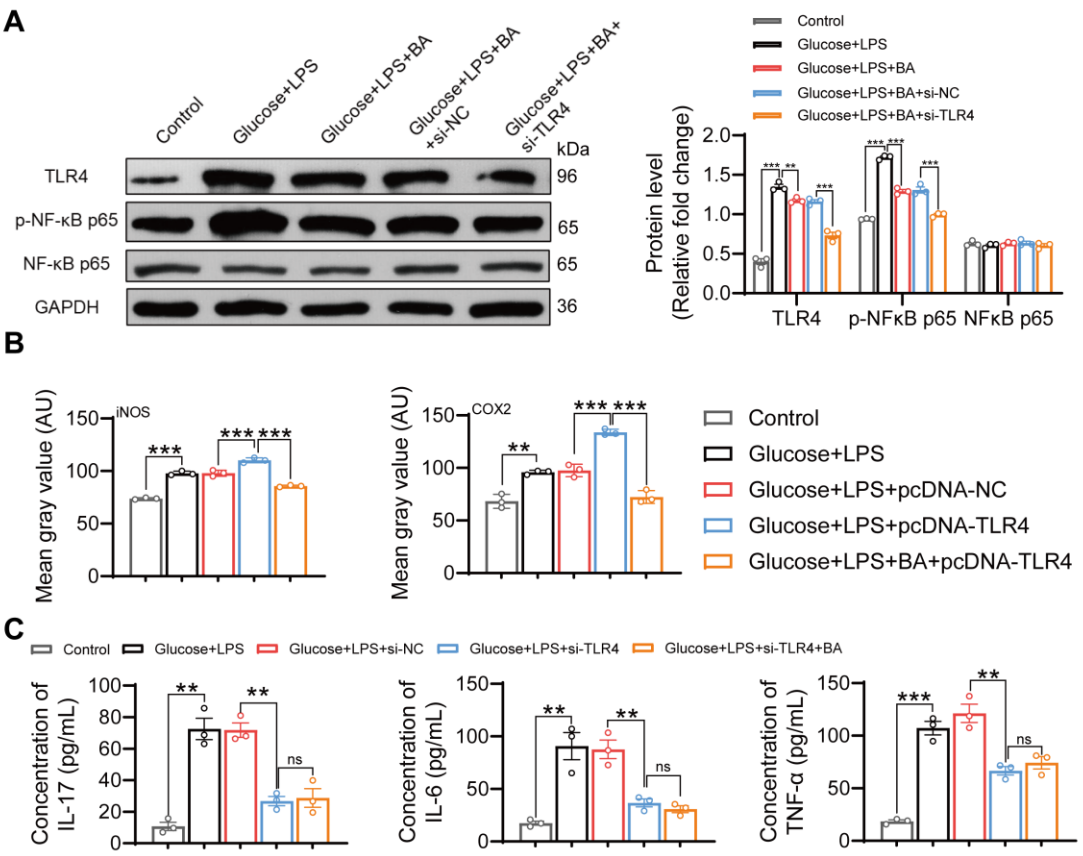

Figure S4. BA reduces inflammation by inhibiting TLR4/NF-κB signaling pathway. Related to Figure 7.

(A) The regulatory effect of TLR4 on NF-κB activation was demonstrated using western blotting. n=3.

(B) Quantification of iNOS and COX2 fluorescence intensity for Figure 7C. n=3.

(C) The concentration of IL-17, IL-6 and TNF-α in the medium of these five groups islet β-cells were tested using ELISA. n=3.

Data represented as means ± SEM. One-way ANOVA for A and C; Two-way ANOVA for B. **P<0.01, ***P<0.001, ns, not significant.

Table S1. Primers used for PCR and qRT-PCR in the study. Related to STAR methods.

Usage	Sequence (5`-3`) Forward	Revese	Product length (bp)
M-Gapdh	TGAAGGGTGGAGCCAAAAG	AGTCTTCTGGGTGGCAGTGAT	227
M-Il-6	TTGCCTTCTTGGGACTGATG	TCATTTCCACGATTTCCCAG	162
M-Il-17	CTCAGACTACCTCAACCGTTCC	ATGTGGTGGTCCAGCTTTCC	141
M-Tnf-α	CTACTCCCAGGTTCTCTTCAAGG	CTCCCAGGTATATGGGCTCATAC	188
M-Ccl3	ACTGCCCTTGCTGTTCTTCTC	GGTTTCTCTTAGTCAGGAAAATGAC	193
M-Ccl8	GCTGCTCATAGCTGTCCCTGT	CCATGTACTCACTGACCCACTTC	227
M-Cxcl2	CTCGCTGTCTGAGAGTTCACTTATT	GTGAATTAGCGAATGCATATCTTT	118
M-Cxcl4	GCGATGGAGATCTTAGCTGTGT	GACATTTAGGCAGCTGATACCTAAC	226
M-Ins1	AGGCAAGTGTTTGGAAACTGC	GCTTGCTGATGGTCTCTGATTAT	190
M-Sry	CGTGGTGAGAGGCACAAGTT	ATGGCATGTGGGTTCCTGTC	282
